# Self reported receipt of care consistent with 32 quality indicators: national population survey of adults aged 50 or more in England

**DOI:** 10.1136/bmj.a957

**Published:** 2008-08-13

**Authors:** Nicholas Steel, Max Bachmann, Susan Maisey, Paul Shekelle, Elizabeth Breeze, Michael Marmot, David Melzer

**Affiliations:** 1 School of Medicine, Health Policy and Practice, University of East Anglia, Norwich NR4 7TJ; 2 RAND Corporation, Santa Monica, CA, USA; 3 Department of Epidemiology and Public Health, University College London; 4 Peninsula Medical School, Exeter

## Abstract

**Objective** To assess the receipt of effective healthcare interventions in England by adults aged 50 or more with serious health conditions.

**Design** National structured survey questionnaire with face to face interviews covering medical panel endorsed quality of care indicators for both publicly and privately provided care.

**Setting** Private households across England.

**Participants** 8688 participants in the English longitudinal study of ageing, of whom 4417 reported diagnoses of one or more of 13 conditions.

**Main outcome measures** Percentage of indicated interventions received by eligible participants for 32 clinical indicators and seven questions on patient centred care, and aggregate scores.

**Results** Participants were eligible for 19 082 items of indicated care. Receipt of indicated care varied substantially by condition. The percentage of indicated care received by eligible participants was highest for ischaemic heart disease (83%, 95% confidence interval 80% to 86%), followed by hearing problems (79%, 77% to 81%), pain management (78%, 73% to 83%), diabetes (74%, 72% to 76%), smoking cessation (74%, 71% to 76%), hypertension (72%, 69% to 76%), stroke (65%, 54% to 76%), depression (64%, 57% to 70%), patient centred care (58%, 57% to 60%), poor vision (58%, 54% to 63%), osteoporosis (53%, 49% to 57%), urinary incontinence (51%, 47% to 54%), falls management (44%, 37% to 51%), osteoarthritis (29%, 26% to 32%), and overall (62%, 62% to 63%). Substantially more indicated care was received for general medical (74%, 73% to 76%) than for geriatric conditions (57%, 55% to 58%), and for conditions included in the general practice pay for performance contract (75%, 73% to 76%) than excluded from it (58%, 56% to 59%).

**Conclusions** Shortfalls in receipt of basic recommended care by adults aged 50 or more with common health conditions in England were most noticeable in areas associated with disability and frailty, but few areas were exempt. Efforts to improve care have substantial scope to achieve better health outcomes and particularly need to include chronic conditions that affect quality of life of older people.

## Introduction

Life expectancy in high income countries has increased by about eight years since 1950, almost half of which can be attributed to health care.^1^ Despite this achievement many opportunities are being missed for health care to improve the quality and length of life. The population of England experiences about 60 000 potentially avoidable deaths annually, which is the highest rate among comparable countries.^2^ Deficits in quality of care have been reported from many high income countries,^3^ ^4^ ^5^ with a comprehensive study on quality in the United States finding that adults received only 55% of recommended care in 1998-2000.^6^

Studies in England have focused mainly on diabetes, heart disease, and asthma, and found widespread deficits in quality of care, although recent studies have reported some improvements.^7^ ^8^ ^9^ National data on the quality of primary care have been reported since 2004 through a scheme to pay general practices according to their performance on the basis of quality indicators in several chronic conditions, and the proportion of included patients for whom the indicators were achieved was 83%.^10^ ^11^ These data are reported by general practices for payment purposes and do not cover conditions excluded from the scheme, secondary care, or care provided in the independent sector. Quality can be substantially worse outside the scheme, with only 36% of indicators in osteoarthritis and depression being achieved, although no comparable national data have been available.^12^

Given the potential of effective health care to improve individual and population health, independent national data on quality of care are needed to inform future initiatives. Most common major health conditions in high income countries occur in middle aged and older people. We assessed the delivery across England of effective healthcare interventions for a wide range of conditions to those aged 50 or more.

## Methods

We collected information on quality of care from the 2004-5 wave of the English longitudinal study of ageing. The sample was selected to be representative of adults aged 50 or more living in private households in England.^13^ It was drawn from households that had previously responded to the health survey for England in 1998, 1999, and 2001. The sample had been drawn from selected postcode sectors and stratified by health authority and deprivation. Interviewers collected data using face to face interviews in participants’ homes.

Questions from the English longitudinal study of ageing on quality of care were derived from quality indicators that were originally developed for the study on assessing care of vulnerable elders, to assess quality of care for conditions chosen according to their prevalence, impact, effectiveness of prevention or treatment, importance in older people, feasibility of measurement, and the potential for quality improvement.^14^ Processes of health care, with evidence linking them to improved outcomes, were measured rather than health outcomes as processes are directly under the control of the healthcare system and not subject to all the other factors that influence health outcomes.^15^ Quality indicators for the conditions that had been assessed in the assessing care of vulnerable elders study using patient interviews were rated for validity in England by an expert panel of clinicians, using an adaptation of the RAND/University of California in Los Angeles appropriateness method for combining clinical evidence with expert opinion.^16^ ^17^ The clinical panel reviewed and scored the degree to which the indicators reflected good practice in the United Kingdom at the time and were advised that the indicators would be used in a national survey. All indicators were intended to assess the delivery of care of a minimum acceptable standard, rather than optimal care. We excluded indicators if the panel disagreed on their validity. Each indicator was classified as suitable for use with adults aged 50 and over or 65 and over. Of the 102 quality indicators that were rated as valid for use in England, 43 were not piloted in the English longitudinal study of ageing, either because of difficulties implementing them in the assessing care of vulnerable elders interview or because they were not a common cause of disability in the health survey for England 2000, and another 27 were not used due to implementation problems identified in the pilots or main survey.

Thirty two indicators for 13 medical conditions were included in the English longitudinal study of ageing: cerebrovascular disease (stroke), depression, diabetes mellitus, falls, hearing problems, hypertension, ischaemic heart disease, osteoarthritis, osteoporosis, pain management, smoking cessation, urinary incontinence, and problems with vision (cataract). We assessed quality mainly for incident conditions to reduce recall bias by asking about relatively recent events. The exceptions, when we assessed quality for prevalent conditions, were diabetes because several of the quality indicators referred to annual assessments that might not have been done if we had assessed incident disease, and hearing problems, osteoporosis, smoking cessation, and vision problems because the quality indicators referred to interventions that were considered more memorable over time.

We classified the quality indicators into one of three domains of care: screening and prevention, diagnosis, and treatment and follow-up. Each of the 13 conditions was classified into one of two clinical categories, general medical or geriatric care, and one of two policy categories, according to whether or not the condition was included in the UK general practice pay for performance contract 2004-5 (see table 1).^10^ Conditions classified within the area of geriatric care were falls, osteoarthritis, urinary incontinence, vision problems (cataract), hearing problems, and osteoporosis. We adapted seven additional questions on the quality of patient centred care from three established survey instruments: the consumer assessment of healthcare providers and systems,^18^ the Foundation for Accountability’s Robert Wood Johnson national strategic indicators survey project,^19^ and the Medicare current beneficiary survey.^20^ Box 1 provides some examples of quality indicators.

**Table 1 tbl1:** Classification of conditions into categories

Condition	General medical	Geriatric	Included in contract*	Excluded from contract*
Cerebrovascular disease	Yes	—	Yes	—
Depression	Yes	—	—	Yes
Diabetes	Yes	—	Yes	—
Falls prevention	—	Yes	—	Yes
Hearing problems	—	Yes	—	Yes
Hypertension	Yes	—	Yes	—
Ischaemic heart disease	Yes	—	Yes	—
Drugs (warfarin)	Yes	—	Yes	—
Osteoarthritis	—	Yes	—	Yes
Osteoporosis	—	Yes	—	Yes
Pain management	Yes	—	—	Yes
Smoking cessation	Yes	—	Yes	—
Urinary incontinence	—	Yes	—	Yes
Vision problems	—	Yes	—	Yes

*UK general practice pay for performance contract 2004-5.^10^

**Box 1 Examples of quality indicators**

**Diabetes mellitus**

- If a person aged 50 or older has diabetes, then his or her glycosylated haemoglobin or fructosamine level should be measured at least annually
- All diabetic persons aged 50 or older should have an annual examination of his/her feet

**Urinary incontinence**

- If a person aged 50 or older has new urinary incontinence that persists for over 1 month or urinary incontinence at the time of a new evaluation, then a dipstick urinalysis and/or mid-stream urine sample should be obtained

**Pain management**

- If a person aged 50 or older has a newly reported chronic painful condition, then treatment should be offered

**Osteoarthritis**

- If oral pharmacological therapy is initiated to treat osteoarthritis among people aged 50 or older, then paracetamol should be the first drug used, unless there is a contraindication to use

We adapted survey questions from the assessing care of vulnerable elders interview for quality indicators that were similar between that study and the English longitudinal study of ageing. The National Centre for Social Research twice piloted the English longitudinal study of ageing computer assisted personal interview instrument.^21^ Survey questions were designed to determine whether participants had an eligible health condition and, if so, whether they had received the indicated health care. Box 2 gives an example of how the survey questions corresponded to the relevant quality indicator. Web extra table C gives a full list of indicators and questions asked and the specifications of the eligible populations. A total of 118 targeted questions was used to assess whether the 32 quality indicators were achieved. Participants were asked questions relevant only to their condition or conditions.

**Box 2 Converting quality indicators to survey questions**

**Diabetes quality indicator**

- If a person aged 50 or older has diabetes, then his or her glycosylated haemoglobin or fructosamine level should be measured at least annually

**Corresponding questions from English longitudinal study of ageing survey**

- Have you ever had a special blood test to see how well your blood sugar was controlled? This test is called a glycated haemoglobin, or haemoglobin A_1c_, or fructosamine. This is a blood test taken at a doctor’s surgery, health centre, or laboratory
- Have you had this test (glycated haemoglobin or fructosamine) done in the past 12 months?

### Statistical analysis

The quality score for each indicator was the number of times that that indicator was achieved divided by the number of times it was triggered, expressed as a percentage, with possible values between 0% and 100%. For example, if 600 participants triggered a quality indicator, and indicated care was given on 500 occasions, the quality score would be 500/600=83.3%. Aggregate quality scores for each condition, for each domain of care and condition category, and overall were similarly calculated from the total number of times indicators in each group were triggered and achieved.^4^ ^22^ We excluded “don’t know” responses from both the numerator and the denominator when calculating quality scores. The data were weighted for non-response and to adjust for respondents’ age and sex distribution to the distribution of the non-institutionalised population from the 2001 census. We took into account the multistage sampling strategy and when relevant multiple indicators per participant using Stata SE 9 svy statistical commands.

## Results

Overall, 10 770 people who had been interviewed at a residential address in England for wave 1 of the English longitudinal study of ageing in 2002 were eligible to participate. Of these, 8688 (80.7%) completed an interview during 2004-5, excluding 92 whose responses were given by a proxy informant. The response rates for the English longitudinal study of ageing had been 67% for wave 1 (2002-3) and 75% for the health survey for England.^21^ In total, 4417 of the wave 2 participants (50.8%) reported having a diagnosis of one or more of 13 study conditions (table 2). The mean age of participants was 66.8 years, and 55.1% were women. The median number of conditions per participant was 1 (range 0-7), with a median of 1 (range 0-14) quality indicator per participant. Participants with at least one condition each had a median of 2 (range 1-14) quality indicators.

**Table 2 tbl2:** Characteristics of 8688 participants for wave 2 of English longitudinal study of ageing interviewed during 2004-5. Values are numbers (percentages) unless stated otherwise

Characteristic	Value
Median (range) age (years)	66 (52 to >90)*
Women	4783 (55.1)
Non-European origin	197 (2.3)
Education (highest qualification):	
Degree or equivalent	1052 (12.1)
No educational qualifications	3411 (39.3)
Median total (range) net household financial wealth (£)	15 987 (−68 000 to 8 000 999)
One or more conditions	4417 (50.8)
Median No (range) of conditions for which participants were eligible†	1 (0 to 7)
Median No (range) of indicators for which participants were eligible†	1 (0 to 14)
Clinical incidence‡:	
Cerebrovascular disease (stroke)	78 (0.9)
Depression	122 (1.4)
Falls	142 (1.6)
Ischaemic heart disease	190 (2.2)
Osteoarthritis	256 (3.0)
Pain management	268 (3.1)
Hypertension	571 (6.6)
Urinary incontinence one year incidence	329 (3.8)
Clinical prevalence:	
Osteoporosis	581 (6.7)
Vision problems (cataract)	594 (6.8)
Diabetes mellitus	705 (8.1)
Hearing problems	1043 (12.0)
Current smoking	1318 (15.2)

£1.00 (€1.3; $1.9).

*All 95 respondents aged more than 90 had exact age withheld from released data.

†Excluding patient centred questions.

‡Incidence rates refer to about two years since previous survey wave unless one year is stated.

Some quality indicators applied to incident conditions and incidence varied from 0.9% reporting stroke (cerebrovascular disease) to 6.6% reporting a new diagnosis of hypertension since the previous survey wave two years previously (table 2). The median time between interviews was 27 months, with 1% of interviews less than two years apart and another 1% more than three years apart. Other indicators applied to prevalent conditions and prevalence varied from 6.7% for osteoporosis to 15.2% reporting being a current smoker.

In all 19 082 opportunities existed for the care indicated by a quality indicator to be delivered. Indicated care was achieved on 11 911 of those opportunities, giving an overall indicator achievement rate of 62.3% (95% confidence interval 61.5% to 63.2%) after adjustment (table 3). The quality of care achieved varied substantially by condition, from 83.0% in ischaemic heart disease to 29.0% in osteoarthritis (table 3). “Don’t know” responses were given by either no participants or one participant for just over half of the indicators and by less than 5% of eligible participants for all indicators except two: 15 participants (19%) responded with don’t know when asked about antihypertensive drugs for stroke (indicator cvd4) and 47 (7%) responded with don’t know when asked about annual measurements for glycated haemoglobin level (indicator diab1; also see web extra table A). Web extra table A gives the full text, individual quality scores and don’t know responses for all 32 quality indicators and seven patient centred questions.

**Table 3 tbl3:** Achievement rates aggregated by condition, adjusted for weighted data, multistage sampling, and multiple indicators per participant

Condition	No of quality indicators	No of times quality indicators achieved	No of times quality indicators eligible	% Quality indicators achieved (95% CI)
Ischaemic heart disease	5	442	530	83.0 (79.7 to 86.4)
Hearing problems	2	1366	1728	78.9 (76.7 to 81.1)
Pain management	1	209	268	77.7 (72.6 to 82.8)
Diabetes	5	1729	2324	74.1 (72.2 to 76.0)
Smoking	1	976	1318	73.5 (71.1 to 76.0)
Hypertension	1	414	571	72.4 (68.6 to 76.2)
Cerebrovascular disease (stroke)	1	51	78	64.9 (53.8 to 75.9)
Depression	3	168	264	63.6 (57.3 to 69.8)
Vision problems	1	339	594	58.4 (54.3 to 62.6)
Osteoporosis	2	399	742	53.3 (49.3 to 57.2)
Urinary incontinence	4	668	1301	50.7 (47.2 to 54.2)
Falls	2	124	284	43.5 (36.5 to 50.6)
Osteoarthritis	4	288	993	29.0 (26.0 to 31.9)
Overall clinical care	32	7173	10 995	65.2 (64.2 to 66.2)
Patient centred care	7	4738	8087	58.4 (56.9 to 59.8)
Overall, including patient centred care	39	11 911	19 082	62.3 (61.5 to 63.2)

Achievement rates for individual quality indicators also varied within a condition—for example, for depression from 45.9% to 78.5%, for ischaemic heart disease from 65.4% to 100.0%, and for osteoarthritis from 17.7% to 35.8%. To expand on the example of ischaemic heart disease, the quality scores for the individual quality indicators for that condition were: 65.4% of those with a new myocardial infarction during the two years since the previous survey wave reported either that a doctor had recommended taking “a medication called a β blocker,” or that they were taking one of the β blockers listed on a card shown by the interviewer; 80.1% of those with new angina or myocardial infarction reported that a doctor had “suggested taking medication to thin the blood, such as warfarin or aspirin, Plavix (clopidogrel), Ticlid (ticlopidine), or other blood thinning medication”; 86.9% of those with high cholesterol levels and either new angina or myocardial infarction reported that a doctor had talked to them about how to lower their cholesterol level (participants were told “this would include changing your diet, losing weight, getting more exercise or taking medication”); 89.1% of participants with angina or a myocardial infarction and who described themselves as current smokers reported that a doctor or nurse had ever advised them to stop smoking; and 100.0% of those with angina or a myocardial infarction and who were taking warfarin had an international normalised ratio test in the past 12 weeks.

Achievement was substantially higher for general medical conditions (74.2%) than for geriatric conditions (56.5%) and was higher for conditions included in the UK general practice pay for performance contract (74.6%) than in excluded conditions (57.7%; table 4). Achievement was also substantially higher for screening and preventive care (79.8%) than for treatment and follow-up care (64.1%), which in turn was higher than diagnostic care (60.0%).

**Table 4 tbl4:** Achievement rates aggregated by condition category and domain, adjusted for weighted data, multistage sampling, and multiple indicators per participant

Variable	No of quality indicators	No of times quality indicators achieved	No of times quality indicators eligible	% Quality indicators achieved (95% CI)
Condition category (clinical):				
General medical	17	3989	5353	74.2 (72.9 to 75.5)
Geriatric	15	3184	5642	56.5 (54.9 to 58.1)
Condition category (policy):				
Included in contract*	13	3612	4821	74.6 (73.2 to 75.9)
Excluded from contract*	19	3561	6174	57.7 (56.3 to 59.2)
Domain:				
Screening and prevention	5	1099	1377	79.8 (77.5 to 82.2)
Diagnosis	6	1387	2299	60.0 (57.7 to 62.2)
Treatment and follow-up	21	4687	7319	64.1 (62.9 to 65.3)

*UK general practice pay for performance contract 2004-5.^10^

Three quality indicators for diabetes and three for ischaemic heart disease in the English longitudinal study of ageing were similar to indicators used in the UK general practice pay for performance contract, and achievement was comparable. For example, haemoglobin A_1C_ levels had been checked in the previous 12 months for 83% of participants in the study compared with 96% in the contract in the previous 15 months; foot checks were received by 83% of patients with diabetes in the study compared with 85% receiving peripheral pulse checks in the contract; and antiplatelet therapy was recommended to 80% of participants in the study in the previous 12 months compared with 92% in the contract in the previous 15 months (table 5). Table 5 also shows the estimated population achievement in the general practice contract, which includes patients “excepted” from the indicator by general practices (M Roland, personal communication, 2006).^11^

**Table 5 tbl5:** Scores for comparable quality indicators in English longitudinal study of ageing (ELSA) 2004-5 and the UK general practice pay for performance contract 2004-5

Condition and ELSA quality indicator* and comparable contract indicator†	Eligible population	Indicator intervention	ELSA and reported contract achievement (%)‡	Estimated contract achievement for population (%)§
Diabetes:				
ELSA diab1	Diagnosed diabetes, aged 50 or more	Measurement of haemoglobin A_1c_ or fructosamine level in past year	83	
Contract DM5	Diagnosed diabetes	Measurement of haemoglobin A_1c_ level in past 15 months	96	93
ELSA diab2	Diagnosed diabetes, aged 50 or more, without renal disease, not receiving angiotensin converting enzyme or angiotensin II receptor blockers	Test for proteinuria in past year	83	
Contract DM13	Diagnosed diabetes	Test for proteinuria in past 15 months	79	71
ELSA diab9	Diagnosed diabetes, aged 50 or more	Foot examination in past year	83	
Contract DM9	Diagnosed diabetes	Peripheral pulse check in past 15 months	85	80
Contract DM10	Diagnosed diabetes	Neuropathy test in past 15 months	84	79
Ischaemic heart disease:				
ELSA ihd2	Diagnosed ischaemic heart disease, aged 50 or more	Antiplatelet therapy	80	
Contract CHD9	Diagnosed ischaemic heart disease	Aspirin or antiplatelet or anticoagulant therapy in past 15 months	92	88
ELSA ihd3	Diagnosed ischaemic heart disease, aged 50 or more, smoker	Counselling for smoking cessation	89	
Contract CHD4	Diagnosed ischaemic heart disease, smoker	Advice on smoking cessation or referral in past 15 months	96	—
ELSA ihd5	Recent myocardial infarction, aged 50 or more	β blocker therapy	65	
Contract CHD10	Diagnosed ischaemic heart disease	β blocker therapy	64	48

*Indicators from ELSA are on bmj.com.

†Indicators in general practice contract are given in full in New General Medical Services contract annex A.^10^

‡General practice contract reported achievement excludes exception reported patients (www.ic.nhs.uk/statistics-and-data-collections/audits-and-performance/qof).

§Includes exception reported patients (M Roland, personal communication, 2006).

## Discussion

Quality of care received by adults aged 50 or more living in private households in England and with a diagnosis of at least one of a range of medical conditions varied substantially by condition, ranging from 83% for ischaemic heart disease to 29% for osteoarthritis. Participants reported better quality of care for general medical conditions than for geriatric conditions and better quality of care for conditions included in the UK general practice pay for performance contract than those excluded.

Strengths of the study include the large number of participants and range of conditions, sampled from across England. The English longitudinal study of ageing includes the independent sector as well as the National Health Service, which is particularly important for interventions commonly provided privately—for example, hip and knee joint replacement, cataract extraction, and provision of hearing aids. The quality indicators were developed through a rigorous process.^16^ Compliance with quality indicators from the assessing care of vulnerable elders study has been associated with longer survival,^23^ quality of care for targeted conditions can be improved by a practice based intervention,^24^ ^25^ and improvements in indicator scores from the assessing care of vulnerable elders study are associated with decreases in healthcare utilisation in older adults.^25^

This study has several limitations. Thirty two quality indicators and seven questions on patient centred care inevitably give an incomplete picture of quality. The quality indicators refer to processes of care rather than to outcomes because of the difficulty attributing individuals’ health outcomes to the quality of their care.^26^ It is arguably more meaningful in this context to know whether they had received care that was likely to be effective than to know their health status, which has many determinants.

Because of the design of the English longitudinal study of ageing the cumulative proportion of responders from the original population sample decreases with each survey wave, and was below half for the second wave analysed in this paper. The data were weighted to correct for non-response, although the age and sex distributions of the weighted and unweighted samples were similar (see web extra table B). Weights cannot account for all differences between responders and non-responders, however, and important differences may exist that we have no information about. For example, participants in the English longitudinal study of ageing may engage better with health services than non-responders. If so this would bias our results towards overestimating the quality of health care received by the population.

The questions were designed for the assessment of quality at population level and some people may be classified as eligible for the quality indicator yet would not have been clinically suitable for the intervention for a particular reason. We made considerable efforts during the development of the survey to target the questions as accurately as possible to the eligible population. Two related sources of potential inaccuracy result from the survey application of quality indicators. Firstly, the translation of quality indicators into a survey resulted in the text of the survey questions not being identical to the indicator text. Secondly, some minor differences were present between the populations specified by quality indicators and the survey populations who were asked the corresponding questions (see web extra table C).

Creating a summary score has benefits and drawbacks.^22^ The purpose in this paper of reporting a summary score was to put these results in the context of other papers^4^ ^6^ ^11^ that report summary scores using equal weighting per indicator. It is plausible that some indicators are somehow more difficult to achieve than others and that some are more important than others. No single accepted method exists for weighting quality indicators for difficulty or importance, although options would include a Delphi-type consensus process and analysis of effectiveness (for example, quality life years gained), and so we weighted all indicators equally. One effect of equal weighting is that conditions affecting more participants than others contributed more to the overall score. Caution is required when comparing quality scores by condition because the selective nature of the indicators included in each condition means that they are not directly comparable.

The data were derived from self reports at interview. The feasibility of measuring quality of care by self report was one of the selection criteria for conditions and quality of care questions for the English longitudinal study of ageing. Concordance between survey self reports and medical records is good for diagnoses of some chronic conditions and process of care measures, including drugs.^27^ Previous work that compared quality measurement using indicators from the assessing care of vulnerable elders study by interview with examination of medical records has shown that self reports tend to score the same or higher than medical records. This suggests that participants either remembered care that had not been documented in their records or reported receiving appropriate care when they had not.^28^ Again, this would have caused us to overestimate the level of quality received rather than underestimate it, although it is also possible that the self reports underestimated quality. Don’t know responses were given by more than 5% of eligible respondents for two indicators: antihypertensive drugs for stroke and annual measurement of glycated haemoglobin levels. The higher rates for don’t know responses may mean that these two indicators are less accurate than others. Both these indicators deal with relatively more technical aspects of care, where patients might be expected to be less knowledgeable.

No one way exists to measure all of quality. Each method measures some portion of quality and has strengths and limitations. In this study a strength was that the English longitudinal study of ageing measured more indicators of quality of care than most UK studies and on a large number of people. The high levels of agreement with similar indicators in the general practice contract 2004-5 provide further validation. Minor differences in scores would be expected from differences in methods, however, as the contract includes adults aged less than 50 and eligibility for indicators is not always identical to the English longitudinal study of ageing (table 5).

These results from the English longitudinal study of ageing are similar to the findings from studies in the United States, which used data extracted from medical records as well as telephone interviews. A US nationally representative survey found that participants received 55% of recommended care in 1998-2000,^6^ and the assessing care of vulnerable elders study, from which the quality indicators for the English longitudinal study of ageing originated, also found a summary quality score of 55% for adults aged 65 or more, with worse care for geriatric conditions.^4^ The assessing care of vulnerable elders study differed from the English longitudinal study of ageing in that its population was vulnerable elders and the number of quality indicators surveyed was higher. The substantial variation in quality between indicators is similar to that found in previous studies from the United States.^4^ ^6^ ^29^ A study from the Netherlands found that guideline recommendations were only followed 67% of the time, again with large variation.^5^ These similarities suggest that compromised quality of care is a property of health systems internationally and that system level changes may be required.^30^

### Implications

The shortfall in achievement of quality indicators reported here has serious implications. Taking coronary heart disease as an example, Gemmell and colleagues have estimated that if 90% of those with coronary heart disease received recommended drug therapy, including β blockers, 3067 coronary events would be prevented annually in England.^31^ This calculation was based on 61% of those eligible receiving β blockers, which is comparable to the 65% who received β blockers in the English longitudinal study of ageing (see web extra table A). If those at high risk of coronary heart disease received better advice on lifestyle, Gemmell and colleagues estimated that 4410 events might be prevented.^31^

Quality for geriatric conditions was relatively poor in this study and no geriatric conditions were included in the general practice contract. It is possible that inclusion of geriatric conditions in future payment for performance schemes would improve quality. It was not possible, however, to determine from this observational study whether the contract had a causal relation with improving quality of care. Care for diabetes and ischaemic heart disease but not all other conditions has been improving for several years.^9^ ^12^ The general medical conditions included stroke, hypertension, diabetes, and ischaemic heart disease, all of which have been subject to major national guidelines such as national service frameworks, and have been the focus of quality improvement activities in primary care trusts.^32^ Arguably barriers to implementing evidence based practice for geriatric conditions are greater, despite the national service framework for older people,^33^ than for the high mortality conditions that are the focus of much medical practice,^34^ ^35^ and the clinical skills required for these conditions may be less well taught to doctors.^4^

Further research is needed to compare these results with quality measurements from directly observed clinical care and from medical records. Longitudinal follow-up will allow analysis of links between the quality of care received by participants and later health outcomes. These data are purely descriptive and further analysis of the data is required to describe participant characteristics, such as socioeconomic status and region of residence, associated with good quality care.^36^

### Conclusion

We found a large gap between recommended care and care that is actually received. What initiatives are needed to improve the quality of health care delivered to people in England? Routine availability of information on performance, used to actively monitor performance, is associated with better care.^37^ In England, performance monitoring through the general practice pay for performance contract has been linked with improved care for included conditions.^9^ ^12^ Making information on performance available for a wider range of conditions is an essential component of quality improvement. Including more conditions that affect the quality of life of older people in future revisions of the general practice contract is one way to do this and has the advantage that the infrastructure is already largely in place, but other solutions should also be sought.

**What is already known on this topic**

Studies of single conditions have suggested shortfalls in quality of care in England
Adults in the United States might receive only about 55% of recommended care

**What this study adds**

Quality of health care for adults aged 50 or more with common health conditions in England varied substantially by condition
Quality scores ranged from 83% for ischaemic heart disease to 29% for osteoarthritis
The greatest scope for improvement is in chronic conditions that affect the quality of life of older people

## Notes

We thank the participants and fieldwork team of the English longitudinal study of ageing. James Nazroo and Martin Roland made useful comments on earlier drafts of this paper.

Ethical approval: London multicentre research ethics committee (MREC/04/2/006).

Provenance and peer review: Not commissioned; externally peer reviewed.

## Notes

**Cite this as:** *BMJ* 2008;337:a957
